# Tomographic reconstruction of ionospheric electron density during the storm of 5-6 August 2011 using multi-source data

**DOI:** 10.1038/srep13042

**Published:** 2015-08-12

**Authors:** Jun Tang, Yibin Yao, Liang Zhang, Jian Kong

**Affiliations:** 1 School of Civil Engineer and Architecture, East China Jiaotong University, Nanchang, 330013, China; 2 School of Geodesy and Geomatics, Wuhan University, Wuhan, 430079, China; 3 Key Laboratory of Geospace Environment and Geodesy, Ministry of Education, Wuhan University, Wuhan, 430079, China; 4 Jiangxi Province Key Lab for Digital Land, East China Institute of Technology, Nanchang, 330013, China

## Abstract

The insufficiency of data is the essential reason for ill-posed problem existed in computerized ionospheric tomography (CIT) technique. Therefore, the method of integrating multi-source data is proposed. Currently, the multiple satellite navigation systems and various ionospheric observing instruments provide abundant data which can be employed to reconstruct ionospheric electron density (IED). In order to improve the vertical resolution of IED, we do research on IED reconstruction by integration of ground-based GPS data, occultation data from the LEO satellite, satellite altimetry data from Jason-1 and Jason-2 and ionosonde data. We used the CIT results to compare with incoherent scatter radar (ISR) observations, and found that the multi-source data fusion was effective and reliable to reconstruct electron density, showing its superiority than CIT with GPS data alone.

CIT technique can reconstruct the 3D spatial and 4D spatiotemporal distribution of IED using the ionospheric delay of GPS signals. It is particularly suitable to monitoring ionospheric structures and variations widely, and known as the low costs on construction and operation of the satellite systems. Thus CIT technique has drawn much attention from ionospheric researchers, and many achievements have been obtained^1^,^2^,^3^,^4^,^5^,^6^,^7^,^8^,^9^,^10^,^11^,^12^. Due to the limitations of observations quantity and geometric structures of GPS signal rays, the inversion function of CIT is ill-posed. Previous studies mainly focused on improving inversion algorithms to solve the ill-posed inversion functions, but they cannot be applied generally, moreover, the ill-posed problem has not been solved radically. To the lower ionosphere, Occhipinti setted up a new method based on the ray-tracing tool TDR to invert the propagation time^13^. For the more complex data-set of CIT, Roy *et al.* developed a new linear regularization tomography method^14^. These methods improved the accuracy of CIT and promoted the development of CIT algorithm. With the establishment of a number of satellite systems and increasing ionospheric observations, the capacity of ionospheric monitoring is enhanced greatly. Observation rays of GPS singnals usually have high elevation angles, the lack of horizontal rays causes relatively lower vertical accuracy in CIT. As early as 1994, Hajj *et al.* proposed to use occultation data for ionospheric tomography^2^, and then successive studies and experiements on ionospheric reconstruction using occultation data have been conducted^15^,^16^,^17^,^18^,^19^,^20^,^21^,^22^. Li *et al.* reconstructed the spatial and temporal distribution of the ionospheric electron density over China, using GPS observations from both ground recievers and low-orbit sattelites of COSMIC^21^. Xiao *et al.* used GPS and CHAMP/GRACE occultation data to reconstruct the spatial distribution of the IED during geomagnetic storms, and the results reflected the ionospheric anomalies well^22^. Zhao *et al*. improved the vertical resolution of reconstructed IED over part of China by adding ionosonde data with GPS data^23^,^24^. Chartier *et al.* regarded ISR observations as background value, and integrated ionosonde and GPS data to improve vertical accuracy of GPS CIT technique^25^.

The above mentioned methods improved the accuracy of ionospheric tomography to some extent, but the insufficient observations and worse vertical accuracy are still unsolved problems. Therefore, we research the reconstruction method by integration of ground-based GPS data, occultation data of the LEO satellite, satellite altimetry data of Jason-1 and Jason-2 and ionosonde data. ISR observations are used as reference to compare and analyze the CIT accuracy of integration method in this study and the method using GPS data alone.

## Multi-Source Data

### Ground-based GPS observations

In recent years, GPS has become an important avenue to monitor ionosphere for its global and continuous observations and high precision. GPS dual-frequency observations are used to calculate the total electron content (TEC) of the ionosphere. The TEC along the propagation path between a GPS satellite and a ground receiver is the combined value of the IED^26^, expressed as:


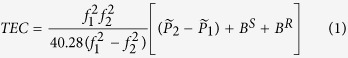


where, 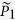 and 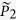 are the dual-frequency pseudo-range observations of the phase smoothing code, *B*^*s*^ and *B*^*R*^ are the satellite and ground receiver for the instrumental biases of the transmitter, respectively. In this process, differential code biases (DCBs) of sattelite and receivers are the largest error, so it is essential to get high-precision DCBs for calculating ionospheric TEC. The DCBs change little in one day, and so can be considered as daily constants^27^.

### Occultation observations

Constellation Observing System for Meteorology, Ionosphere, and Climate (COSMIC) is one of the primary operating occulatation system. It includes COSMIC-I and COSMIC-II projects, the low-earth orbit satellites carry GPS receivers, which record GPS dual-frequency carrier phase sequences with sub-millimeter precise and highly sampling frequency. There are about 2,000 occultation events worldwide in one day. The COSMIC observations are used to produce atmospheric profiles with high resolution and high accuracy. The IonPrf product, provided by the COSMIC data analysis center CDAAC, gives vertical IED profiles, ranging from the bottom of the ionosphere to the satellite orbital altitude.

### Satellite altimetry observations

Jason-1 and Jason-2 are the main ocean altimetry satellites to measure global sea-level changes. They carry the radar altimeters with the dorminant frequency of Ku-band and auxiliary frequency of C-band. Jason 1 and 2 have the orbit with coverage of 66°N-66°S, and altitude of 1336 km. Jason-2 is the next generation NASA ocean altimetry mission which will be the follow on to Jason-1. The two satellites fly on the opposite part of the Earth. The impact of ionosphere on the propagation path of electromagnetic waves is proportional to the square of electron density, and is inversely proportional to the square of the electromagnetic wave frequencies^28^, thus the vertical TEC (VTEC) from altimeter is calculated as:


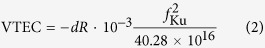


Where, *f*_Ku_ is the Ku-band carrier frequency in Hz. Radar altimeter can directly obtain the differential group path of transmitted signal. The ionospheric range delay 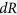 derived from the altimeter measurements at the two frequencies is directly provided in mm, and has to be transformed into TECU according to 
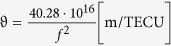
. 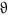 is the factor, it gives the ionospheric path delay in meter per one TECU, related to a certain frequency *f* in Hz. Altimetry satellite can directly obtain the ionospheric TEC in the vertical direction, and especialy has higher credibility in the lower latitude and equatorial regions. The errors of altimeter VTEC are usually less than 3 TECU (1 TECU = 10^16^ el/m^3^) caused by the instrument hardware, satellite attitude, ocean waves and other factors. Specially, VTEC obtained by altimeters cannot include that above the satellite orbit, so it is smaller than the VTEC obtained from GPS. However, the plasma electron density at higher altitude is quite small, causing the error even less than systematic error, therefore this error is neglected^29^.

### Ionosonde observations

Ionosonde is a specialized radar system for examination of the ionosphere, it emits upward high-frequency radio pulses with the frequency range of 1 ~ 30 MHz. By receiving reflected waves from different ionospheric layers and recording the transmitted time, the ionogram is produced. When the transmitted frequency equals ionospheric plasma frequency *f*_*p*_, the pulse will be reflected back, and the ionospheric plasma frequency *f*_*p*_ can be expressed by IED. With the different reflection frequencies, electron density at different altitudes can be obtained. For the pulse with higher frequency, the transmitted signal will not be reflected, but directly pass through the ionosphere. Ionosphere has several layers, which are reflected with clear traces on the ionogram. The maximum electron density of each layer corresponds to the maximum plasma frequency of the layer^30^.


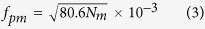


Where, 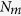 is the maximum electron density in unit of el/m^3^, *f*_*pm*_ is the maximum frequency that can be reflected by the layer, i.e. critical frequency, denoted by *f*_0_ (e.g. *f*_0_E and *f*_0_F represent critical frequency of E layer and F layer, respectively), in units of MHz.

## Method

### Ionospheric single layer model

We fit VTEC obtained from different observations in above section with the spherical harmonic model. The expression of spherical harmonic model is as follows^31^:


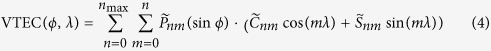


Where, *ϕ* is geomagnetic latitude of ionospheric puncture point, *λ* is solar hour angle of the puncture point in solar fixed coordinate system, *n*_max_ is the maximum degree of spherical harmonic function, 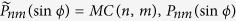 is *n* degree *m* order naturalization Legendre function, *MC*(*n*, *m*) is Naturalization function, *P*_*nm*_(sin*ϕ*) is classic Legendre function, 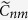 and 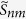 are the unknown spherical harmonic expansion coefficients.

In this paper, the method of normal equation superposition is used to model ionospheric VTEC. Parameters to be resolved include spherical harmonic coefficients, DCBs of receivers and satellites, systematic deviations of altimeter and occultation observations. Considering that the precisions of data from ground-based GPS, satellite altimetry and occultation are not consistent, this study assigns their weights using the Helmert variance component estimation (HVCE) method. The estimation formula can be written as^32^:


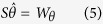


Where the specific forms of *S*, 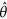, and *W*_*θ*_ can be found in [32]. The formula of HVCE is very complex, matrix inversion is needed after a continuous matrix multiplication. Then, either of the following approximate formulas are often in the actual calculation:


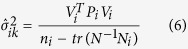


Where, the subscript of 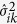 stands for the *k* th iteration of the *i* th variance component, *V*_*i*_ are the residual vector of the measurements corresponding to the design matrix of the measurements corresponding to the variance component, *P*_*i*_ are the weight matrices of the corresponding types of measurements, 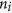 is the number of the components of *V*_*i*_, *N* is the normal equation matrix.

### Three-dimensional CIT model

We assume that the IED of each pixel is constant in the inversion region. Each set of TEC values along the propagation path from a satellite to a receiver can be expressed as:


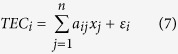


where, 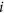 is the number of the ray, *x*_*j*_ is the IED in pixel *j*, *α*_*ij*_ denotes the length of the 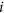
th ray path through pixel *J*, *n* represents the number of all the pixels, and *ε*_*i*_ is the observation noise of the *I* th ray path. Equation (7) can be further written in matrix form:


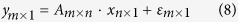


Where, *m* is the number of TEC measurements, *y* is a column vector of the *m* with the absolute TEC from GPS observations, *A* is an *m* × *n* matrix corresponding to the discrete grid, *x* is a column vector of the *n* with electron density at each voxel, and *ε* is the noise.

In this study, GPS data, occultation data, Jason-1 and Jason-2 satellite altimetry data and ionosonde data are integrated to reconstruct IED, the inversion expression is:


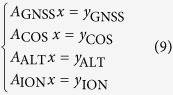


Where, *y*_GNSS_, *y*_COS_, *y*_ALT_ and *y*_ION_ represent TEC obtained from ground-based GNSS observations, occultation observations, altimetry satellites observations and ionosonde observations respectively, *A*_GNSS_, A_COS_, *A*_ALT_ and *A*_ION_ represent the corresponding coefficient matrix, *x* represents the electron density to be estimated.

### Experiments and Analysis

#### Geomagnetic activity

**Figure 1:**
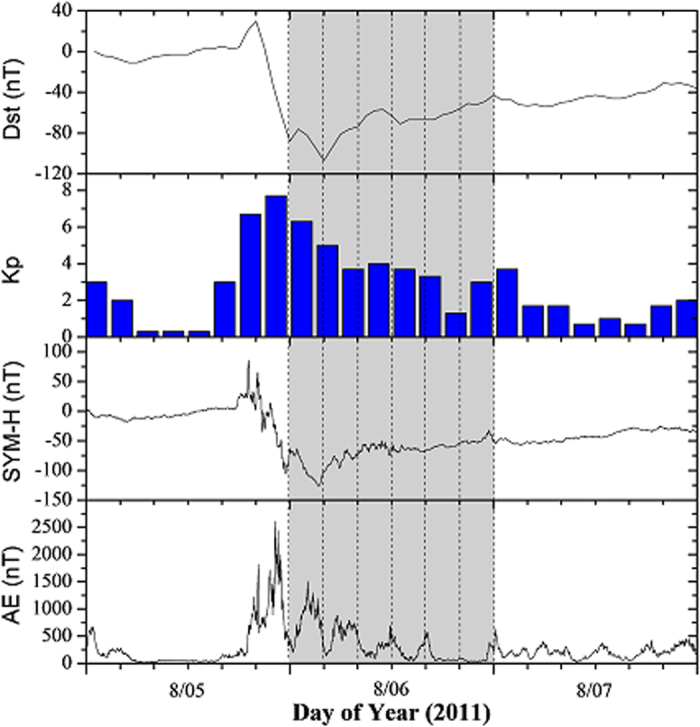
Geomagnetic activity index during 5-7 August 2011.

A moderate geomagnetic storm occurred on 5–6 August 2011. Figure 1 shows the Dst index, planetary radiation index Kp, symmetrical ring current index SYM-H and polar electrojet index AE during 5–7 August 2011. From the variation of Dst we can see that the storm was a sudden commencement storm. The sudden storm commencement (SSC) occurred at 17:00 UT on 5 August 2011, at 19:00 UT the Dst index reached the maximum value, then rapidly declined, and reached the minimum value of −107 nT at 3:00 UT on 6 August, at which the Kp index was 5+. SYM-H index reached the minimum of -126 nT at about 3:25 UT on 6 August. AE index experienced twice rapidly increase during the main phase, indicating a large amount of solar energy injected into the polar ionosphere. After that, the geomagnetic storm came into the recovery phase.

#### Data Sources

To conduct the CIT based on the above integration method, this study reconstructs the 3D IED distribution during the geomagnetic storm on August 6 2011 using the integration method, the study area covers the range of longitude 100°W ~ 30°W, latitude 40°S ~ 50°N and altitude 100 km ~ 1000 km. The reconstructed IED maps have the horizontal resolution of 2° and altitudinal resolution of 50 km. In this paper, we use the method described by Yao *et al.* to reconstruct the 3D IED distribution^33^. The method has used total variation minimization in three dimensions combined with algebraic reconstruction technique.

**Figure 2:**
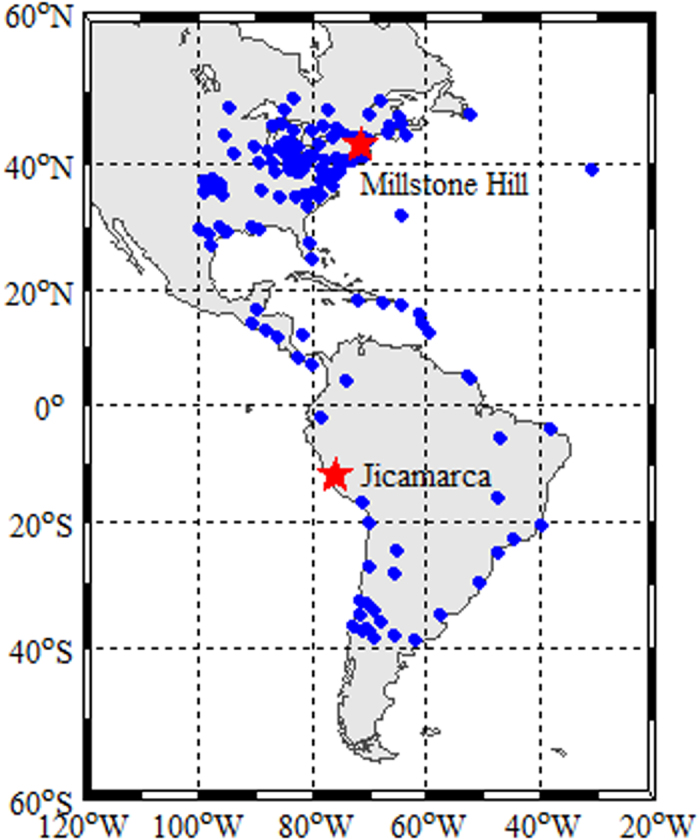
Distribution of GPS and ISR sites, 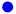 represent GNSS stations 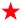 represent ISR sites. This figure is drawn using Matlab software.

The ground-based GNSS observations in this study come from IGS sites. ISR data from Jicamarca (76°W, 11.9°S) and Millstone Hill (71.5°W, 42.6°N) observations were used as reference to do independent examination. Station Distribution is showed in Fig. 2.

**Figure 3:**
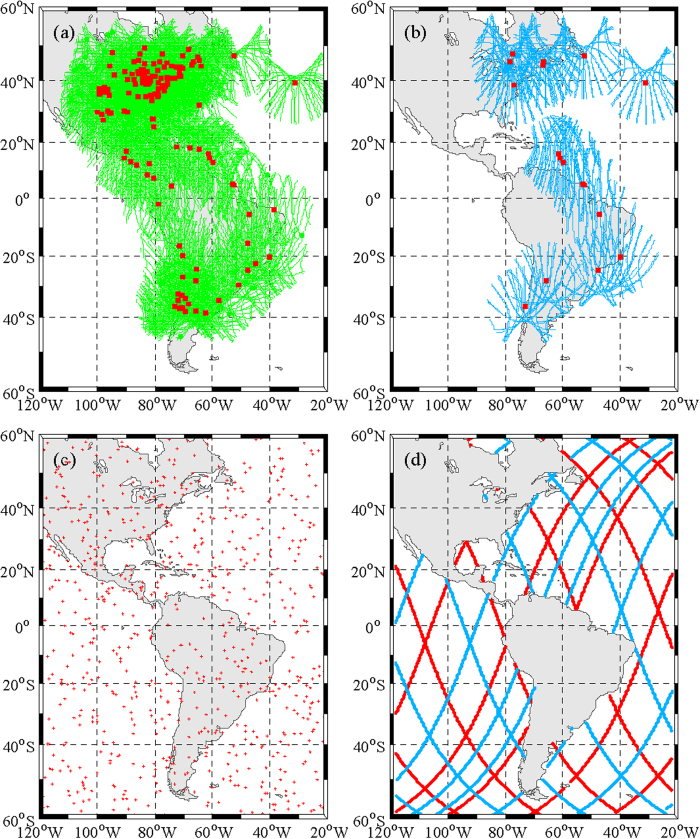
Distribution of multi-source data in the research region on 6 August, 2011. This figure is drawn using Matlab software.

Figure 3 shows the distribution of multi-source data in the research region selected on 6 August 2011. Figure 3(a) show the GPS and GLONASS IPP tracks at altitude of 450 km. The red squares which is in the middle of the trace of IPP stand for the space location of the GPS and GLONASS stations. Figure 3(c) shows the distribution of COSMIC occultation events. The red dots indicate the location of the tangent points during these setting occultations. Figure 3(d) shows the trajectories of Jason satellites, in which the red lines represent Jason-1 trajectory and blue lines represent Jason-2 trajectory. From Fig. 3, we can see that there are plenty of data covering the research region by integrating observations from the different observing systems, which is benefit for ionospheric tomography.

#### Experiments

**Figure 4:**
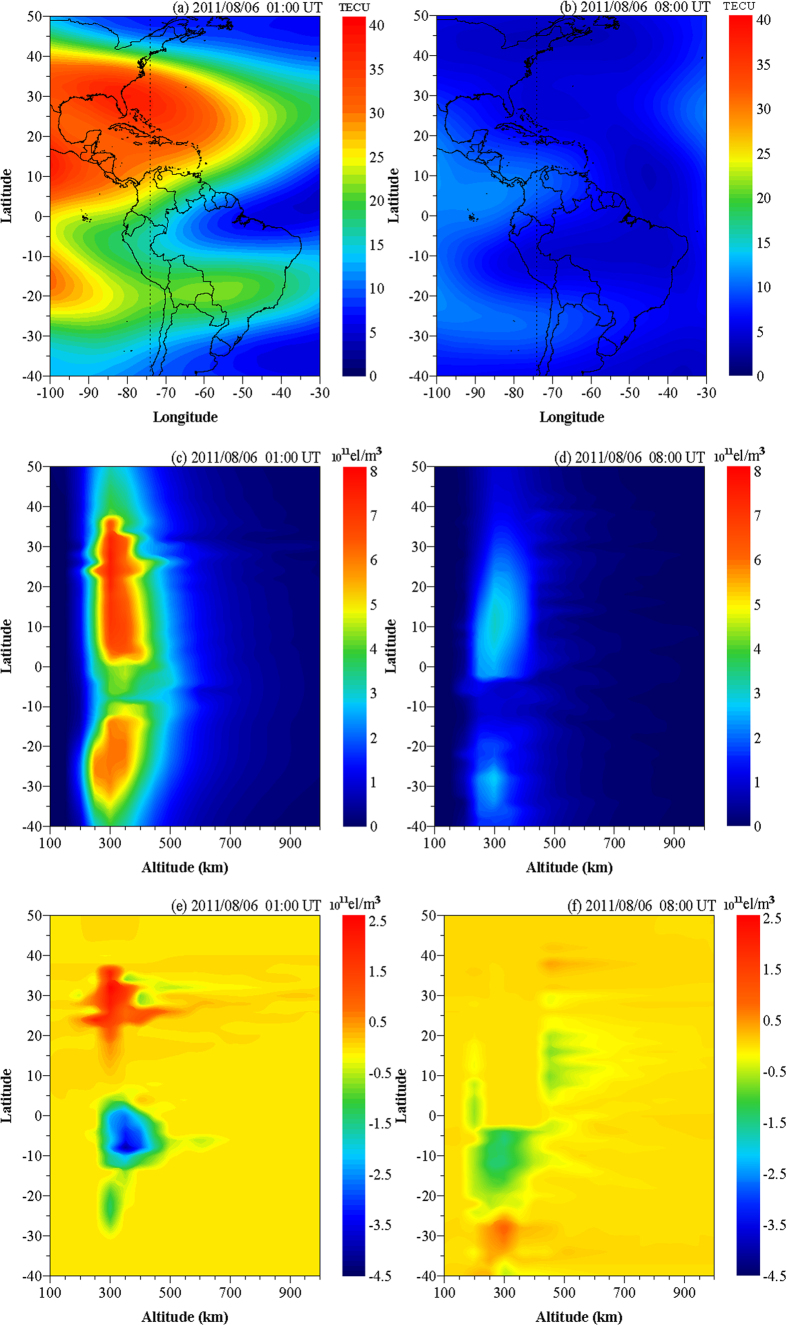
TEC and IED reconstruction maps at 01:00 UT and 08:00 UT on 6 August 2011 ((**a**) and (**b**) are TEC maps, (**c**) and (**d**) are IED distributions, (**e**) and (**f**) are difference distributions). This figure is drawn using Surfer software.

To examine the feasibility of CIT based on multi-source data, we integrate observations from the four systems to reconstruct the three-dimensional distribution of IED during the geomagnetic storm occurred on 6 August 2011. Figure 4(a) are TEC maps at 01:00 UT and 08:00 UT on 6 August 2011 respectively. Figure 4(c), 4 (d) show the electron density distribution, reconstructed by multi-source data, at 74°W at 01:00 UT and 08:00 UT on 6 August 2011 respectively. Figure 4(e) show the electron density distribution of difference which is between the reconstructed values and the values form IRI model, at 74°W at 01:00 UT and 08:00 UT on 6 August 2011, respectively. Comparing Fig. 4(a) with 4 (c), we can see that IEDs have bigger values at two regions of north and south hemispheres. Figure 4(c) shows an ionospheric trough at about 5°S, which is consistent with the TEC distribution of Fig. 4(a). Figure 4(b) have the similar distribution characters, and the ionospheric trough in Fig. 4(d) locates at about 10°S. The good agreement between TEC map and IED distribution verifies that multi-source data integration method proposed in this study is correct and applicable. Figure 4(e) shows that extreme negative anomaly disturbance appears in about 5°S at altitude of about 350 km and extreme positive anomaly disturbance appears in about 20°N to 35°N at altitude of about 300 km. Figure 4(f) shows that extreme negative anomaly disturbance appears in about 10°S at altitude of about 300 km. These negative anomaly disturbances appear in the location of ionospheric trough. They are the large scale perturbations.

Tables 1 and 2 provide the inversion results of the error statistics of the two methods at Jicamarca and Millstone Hill stations, respectively. From the two tables, it can be seen that the reconstruction errors of diverse data are less than those of only GPS data as a whole. Thus, we confirm the validity and superiority of CIT with diverse data.

**Figure 5:**
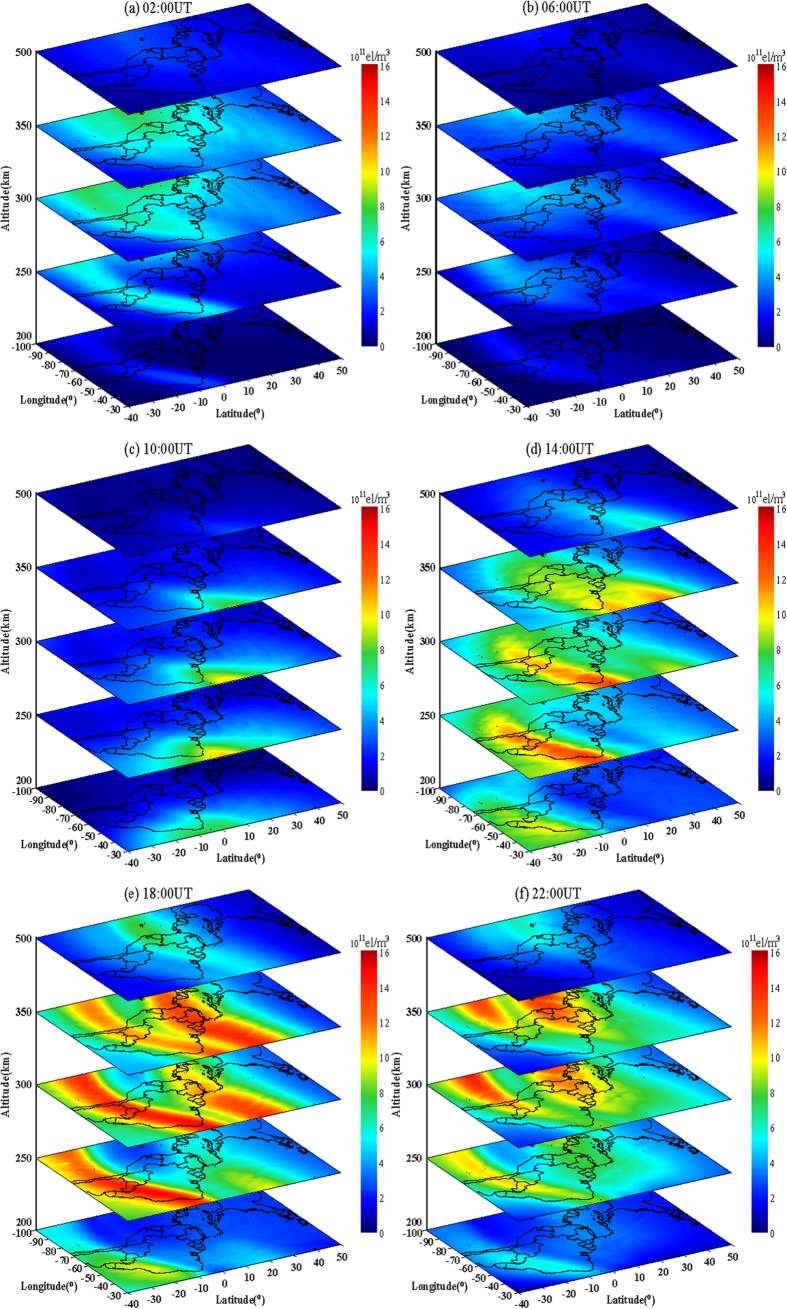
Reconstructed IED distribution at different altitudes. This figure is drawn using Surfer software.

Figure 5 shows the IED maps at different altitudes reconstructed by integration of multi-source data. As can be seen, the electron density reaches maximum value at about 250 km to 300 km, which should be the ionospheric F2 layer. At each same altitude, IEDs decrease from 02:00 UT to 06:00 UT, and have larger values in the west than in the east. IEDs increase from 06:00 UT to 18:00 UT, and have larger values in the east. From 18:00 UT to 22:00 UT, IEDs decrease again, and the electron density in the west are higher than those in the east. IEDs are larger near the equator than at high latitudes.

**Figure 6:**
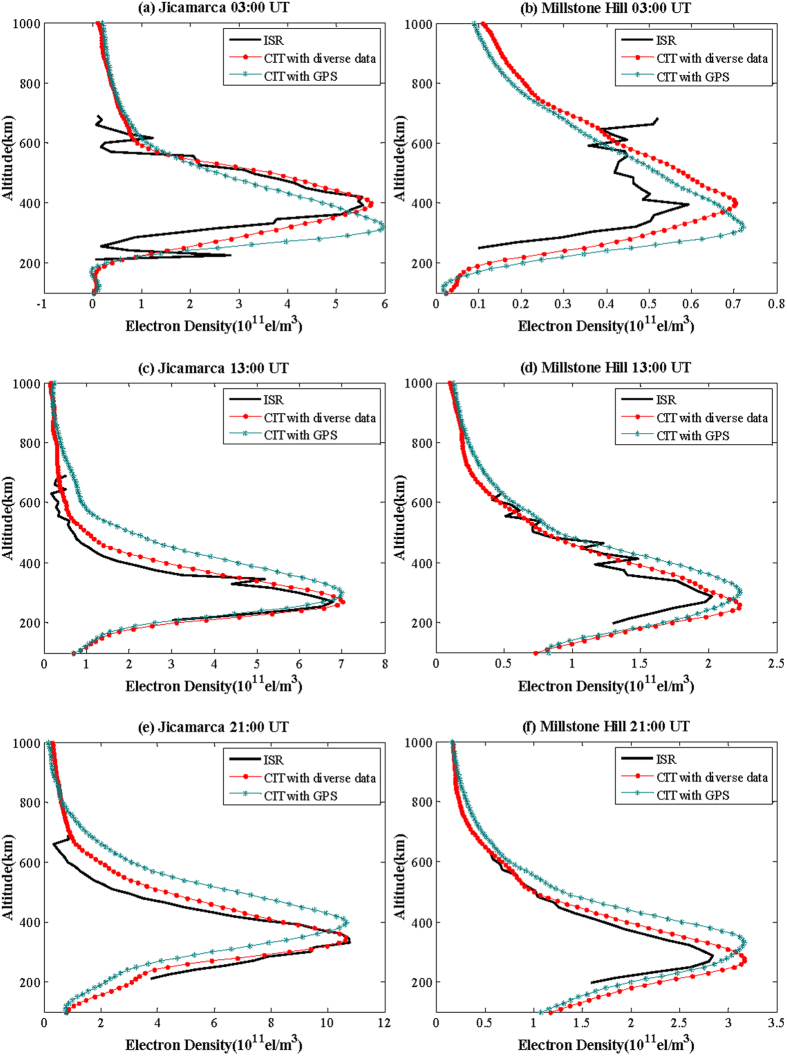
Comparison of IED profiles obtained from multi-source data CIT, GPS data CIT and ISR observations ((**a**) and (**b**) are at 03:00 UT, (**c**) and (**d**) are at 13:00 UT, (**e**) and (**f**) are at 21:00 UT).

Figure 6 shows the comparison of electron density profiles obtained from multi-source data ionospheric tomography, GPS data alone ionospheric tomography and ISR observations. This figure shows clearly that we arrive to reproduce the large scale dynamics but not the short scale perturbations. Form Fig. 4(e),(f) and 6, they show the large scale perturbations. The Figure 6(a) are profiles at 03:00 UT of Jicamarca and Millstone Hill sites respectively. The Fig. 6(c) are profiles at 13:00 UT and Fig. 6(e) are profiles at 21:00 UT of the two sites. The comparison result indicates that for the two different ionospheric tomography methods, profiles of multi-source data are overall closer to the ISR profiles, showing higher vertical accuracy of multi-source data tomography. The experiments have verified the reliability and superiority of CIT using the multi-source data.

## Conclusions

Because the uneven distribution of ground GNSS stations and limited observing angle, CIT with only ground-based GPS data has severely ill-posed problem and the reconstructed IEDs have poor vertical accuracy. In this paper, ground-based GPS data, occultation data of the COSMIC, satellite altimetry data of Jason-1 and Jason-2 and ionosonde data are integrated to conduct CIT and to study ionospheric variation, the IED distributions are reconstructed at both north and south hemispheres during a geomagnetic storm. The result shows the correctness and reliability of CIT based on multi-source data. To further test the method, ISR observations are used to compare and analyze the accuracy of CIT based on multi-source data and CIT based on GPS data. The comparison result indicates that integration of observations from the four systems is able to improve the accuracy of CIT model. Observations of longer period will be helpful for better understanding both regional and global variations of ionosphere. The present results suggest the method proposed by us is applicable in ionospheric tomography. The physical mechanism of ionospheric disturbances also need further research during magnetic storms.

## Additional Information

**How to cite this article**: Tang, J. *et al.* Tomographic reconstruction of ionospheric electron density during the storm of 5-6 August 2011 using multi-source data. *Sci. Rep.* **5**, 13042; doi: 10.1038/srep13042 (2015).

## Figures and Tables

**Table 1:** Errors of NmF2 and hmF2 reconstruction using the two methods at Jicamarca station.

	CIT with diverse data		CIT with GPS	
	NmF2(10^11^el/m^3^)	hmF2(km)	NmF2(10^11^el/m^3^)	hmF2(km)
Maximum absolute error	0.46	84.7	0.57	140.4
Average absolute error	0.19	23.4	0.25	60.5
RMSE	0.27	31.3	0.44	71.0

**Table 2:** Errors of NmF2 and hmF2 reconstruction using the two methods at Millstone Hill station.

	CIT with diverse data		CIT with GPS	
	NmF2(10^11^el/m^3^)	hmF2(km)	NmF2(10^11^el/m^3^)	hmF2(km)
Maximum absolute error	0.66	97.5	0.80	150.3
Average absolute error	0.33	20.2	0.48	31.4
RMSE	0.37	36.7	0.51	42.6
